# Controlled Thin Polydimethylsiloxane Membrane with Small and Large Micropores for Enhanced Attachment and Detachment of the Cell Sheet

**DOI:** 10.3390/membranes12070688

**Published:** 2022-07-03

**Authors:** Yeongseok Jang, Hyojae Kim, Jinmu Jung, Jonghyun Oh

**Affiliations:** 1 Department of Mechanical Design Engineering, College of Engineering, Jeonbuk National University, Jeonju 54896, Korea; ysjang@jbnu.ac.kr; 2 Center for Social Innovation Policy, Office of S&T Policy Planning, Korea Institute of S&T Evaluation and Planning, Eumseong 27740, Korea; hyojaekim@kistep.re.kr; 3 Department of Nano-bio Mechanical System Engineering, College of Engineering, Jeonbuk National University, Jeonju 54896, Korea

**Keywords:** micropore, membrane, polydimethylsiloxane, cell sheet, detachment

## Abstract

Polydimethylsiloxane (PDMS) membranes can allow the precise control of well-defined micropore generation. A PDMS solution was mixed with a Rushton impeller to generate a large number of microbubbles. The mixed solution was spin-coated on silicon wafer to control the membrane thickness. The microbubbles caused the generation of a large number of small and large micropores in the PDMS membranes with decreased membrane thickness. The morphology of the thinner porous PDMS membrane induced higher values of roughness, Young’s modulus, contact angle, and air permeability. At day 7, the viability of cells on the porous PDMS membranes fabricated at the spin-coating speed of 5000 rpm was the highest (more than 98%) due to their internal networking structure and surface properties. These characteristics closely correlated with the increased formation of actin stress fibers and migration of keratinocyte cells, resulting in enhanced physical connection of actin stress fibers of neighboring cells throughout the discontinuous adherent junctions. The intact detachment of a cell sheet attached to a porous PDMS membrane was demonstrated. Therefore, PDMS has a great potential for enhancing the formation of cell sheets in regenerative medicine.

## 1. Introduction

A porous membrane can be defined as a matrix composed of nano- and micro-scaled pores or holes. The choice of membrane material and fabrication technique has a significant influence on the formation of controlled pores, which are required for their target applications such as separation, membrane distillation, fuel cell, and biomedical applications [1,2,3,4,5]. In addition, the performance of the fabricated membrane is dependent on the size and number of the controlled pores [6,7,8]. The infinite diversity and customization possibilities of porous membranes have continued to expand their application areas in various ways.

One of the most promising biomedical applications of porous membranes is the formation of a cell sheet using membranes with micro- and nanopores for the regeneration of damaged tissues caused by diseases or accidents [9,10,11,12,13]. To supply water from a cell sheet periphery, temperature-responsive Poly(N-Isopropylacrylamide) (PIPAAm)-porous membranes (mean roughness: 4.40 nm) have been developed for complete cell sheet detachment [14,15,16]. Cell sheets were cultured on cell culture inserts with porous polyethylene terephthalate track-etched membranes (membrane pore size: 1 μm) to overcome the diffusion limit [17]. A track-etched porous polycarbonate membrane (thickness: 25 μm, pore size: 0.4 μm, pore density: 1.6 × 10^6^ pores/cm^2^) on which the epithelial cell sheets were attached was employed to allow feeder cells to supply nutrition to the epithelial cells [18]. The electrochemical cell detachment approach was applied to a porous membrane substrate (pore size: 0.4 μm, pore density: 1.0 × 10^8^ pores/cm^2^) (BD Biosciences, Franklin Lakes, NJ, USA) to provide pathways for the culture medium during cell sheet detachment [19]. Stem cell sheets were supported by an electrospun gelatin/chitosan nanofibrous membrane encapsulating vascular endothelial growth factor plasmids for plasmid delivery and cell activation to accelerate wound healing [20]. Polyethylene glycol diacrylate through-hole membranes (hole size: 200 μm) functioned as the molecular transport across a hydrogel layer and enabled time-controlled delamination without damaging the capillary network [21]. Poly(l-lactic acid-co-ԑ-caprolactone) electrospun membranes were applied to develop a perfusable engineered vascular network, resulting in more cell proliferation and variation in the shear-stress-related genes expression [22]. A human-induced pluripotent stem-cell-derived airway epithelial cell sheet cultured on a polyethylene terephthalate membrane (pore size: 3 μm, pore density: 2.0 × 10^8^ pores/cm^2^) (Corning #353292, Castle Rock, CO, USA) was transplanted into the middle ear of rats [23].

As shown above, it is challenging to freely tune the membrane thickness and pore size to optimize membranes for the target applications. The substrate in which the cells are cultured requires a porous structure that provides the smooth circulation of cell media as well as mechanical properties as a support for cell growth. Therefore, it is necessary to introduce polydimethylsiloxane (PDMS), a biomaterial showing potential as a porous polymeric membrane. PDMS-based membranes allow the precise control of well-defined pores through the combinative interaction of material, morphology, and fabrication technique [5,24,25]. In addition, the roughness and softness of PDMS caused by surface pores enable cells to directly and actively adhere on the PDMS membrane, which has tunable transport properties, unlike the extracellular matrix, where collagen, fibronectin, and laminin are needed to induce the passive adhesion of cells [26,27,28]. Moreover, thin porous membranes with both small and large pores play an important role in cell growth and tissue formation on the substrate of cell sheets.

In this paper, we introduce a novel approach to manufacturing controlled microporous PDMS membranes suitable for the formation of cell sheets. First, microbubbles generated in a PDMS solution were controlled by the degree of mixing using a Rushton impeller. Then, the thickness of the PDMS membranes was tuned by spin-coating speed. The morphology of the porous PDMS membrane could be determined by optimizing a combination of mixing and spin-coating of the PDMS solution. The mechanical and chemical properties of the fabricated porous PDMS membranes were measured. The cellular performance of the porous PDMS membranes was evaluated through the viability and active adhesion of cells cultured on the membranes. The intact detachment of a cell sheet attached to a porous PDMS membrane was demonstrated, indicating the suitability of this method for tissue regeneration applications.

## 2. Materials and Methods

### 2.1. Design of Modified Rushton Impeller for PDMS Mixing

**Figure 1:**
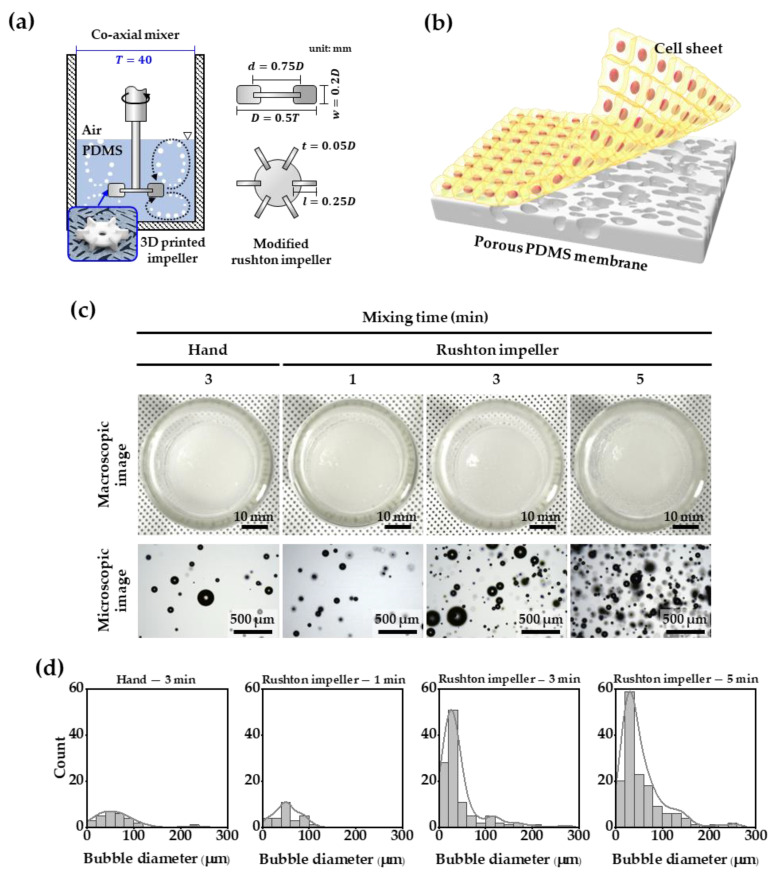
(**a**) Schematic illustration of the Rushton impeller mounted on a co-axial mixer, (**b**) schematic of cell sheet on porous PDMS membrane, (**c**) macroscopic and microscopic images of bubbles generated by hand and Rushton mixing, and (**d**) quantitative measurement of microbubbles in PDMS solution.

A Rushton impeller was used for effective air bubble mixing to make a porous PDMS membrane. The Rushton impeller is an excellent blade for mixing gas/liquid phases. We modified the parameters of the well-known Rushton impeller design [29,30,31] to suit our co-axial mixer, as shown in Figure 1a. The diameter of the mixing chamber was T = 40 mm. Accordingly, the outer diameter, D, of the Rushton impeller was 20 mm; the disc diameter, d, was 15 mm; blade length, l, was 5 mm; blade height, w, was 4 mm. The thickness, *t*, of the modified Rushton impeller was set to 1 mm in consideration of the machinability of the stereolithography 3D printer. The modeled Rushton impeller was printed using a Form 2 printer (Formlabs Inc., Somerville, MA, USA) with a layer thickness of 0.05 mm, by adding supporters with a touchpoint size of 0.4 mm in PreForm software (ver. 3.17.0, Formlabs Inc., Somerville, MA, USA). The printed modified Rushton impeller was washed with isopropanol and then UV cured at 60 °C for 15 min using Form Cure (Formlabs Inc., Somerville, MA, USA).

### 2.2. Fabrication of PDMS Membrane

A PDMS solution containing 3 g of silicone elastomer base and 0.3 g of curing agent (Sylgard 184, Dow Corning Corp., Midland, MI, USA) was mixed for 1, 3, and 5 min using a 3D-printed Rushton impeller mounted in a co-axial mixer. For the control membrane, a mixed solution was spin-coated on silicon wafer (4 inch, Silicon Technology Corporation, Saku, Japan) at 5000 rpm for 60 s, and was degassed under vacuum for 30 min, followed by thermal curing at 120 °C for 1 h. To fabricate four different porous membranes, the mixed solution was spin-coated on silicon wafer at different spin speeds (2000, 3000, 4000, or 5000 rpm) for 30 s without degassing. The uncured coating layer was steamed at 120 °C and 0.12 MPa for 20 min.

### 2.3. Observation of the Five Different Membranes Structures

The morphology of the five different membranes was visualized using a field-emission scanning electron microscope (FE-SEM; SUPRA 40VP, CarlZeiss AG, Oberkochen, Germany) at a 5 kV accelerating voltage. The top, bottom, and cross-section of the membranes were sputtered with a 10 nm Pt coating, and then FE-SEM images were taken. The pore size distributions were determined using the threshold function with ImageJ software (ver. 1.53f accessed on 25 October 2020, National Institutes of Health, MD, USA, https://imagej.nih.gov/ij).

### 2.4. Characterization of Control and Porous Membranes

The mechanical properties of the membranes were evaluated by measuring the Young’s modulus and roughness using an atomic force microscope (AFM) (XE-100, Park Systems Corp., Suwon, South Korea). The force–distance curves were obtained at a constant force of 0.1 μN with an approach speed of 0.3 μm/s for pushing and releasing from the surface. Data analysis was performed using XEI software (ver. 4.3.4, Park Systems Corp., Suwon, South Korea), and the Young’s modulus was calculated using the Hertz model (*n* = 10). The surface topographical structure was scanned at a rate of 0.3 Hz in tapping mode within a square of 40 × 40 μm, and was reconstructed for 2D and 3D views. Simultaneously, the mean roughness was evaluated from the scanned area.

The chemical properties of the control and the porous membrane surfaces were analyzed using an attenuated total reflectance Fourier transform infrared spectroscopy (ATR-FTIR) (Frontier, Perkin Elmer, MA, USA). The measurements were taken in random areas at the same pressure. FTIR spectra was recorded from 4000 to 400 cm^−1^ at a 1 cm^−1^ resolution.

To confirm the difference in hydrophobicity between the control and porous membranes, the contact angles of the membrane surfaces were measured (Phoenix 300 Touch, Surface Electro-Optics, Suwon, Korea).

### 2.5. Cell Culture

Adult normal human primary epidermal keratinocytes were obtained from ATCC (PCS-200-011™), cultured in a KGM-Gold BulletKit (Lonza, Switzerland), using growth supplements (ATCC, PCS-200-040) with 1% penicillin–streptomycin–amphotericin (ATCC-PCS-999-002). All cell cultures were in a humidified atmosphere of 5% CO_2_ at 37 °C, and the culture media were refreshed every 2–3 days. Before the keratinocytes were seeded on the membranes, the membranes were sterilized in an autoclave at 121 °C. The sterile membranes were treated with plasma to improve cell adhesion. The plasma-treated membrane was cut to a diameter of 13 mm, and fixed to a 35 mm confocal dish (100350, SPL life sciences Co., Ltd., Pocheon, South Korea) with a 13 mm diameter hole. The keratinocytes (passage 2) were seeded on respective membranes at 50,000 cells/cm^2^ and cultured for 7 days. After the 7 days, the generating cell sheet was carefully touched using a sterile tweezer at the edge of the membrane, and the culture plate was gently shaken to separate the cell sheet, as shown in Figure 1b.

### 2.6. Immunofluorescent Staining

The cell attachment and viability were estimated using a live/dead viability/cytotoxicity kit (Thermo Fisher Scientific, Waltham, MA, USA). The keratinocytes were pre-cultured for 1, 3, or 7 days, and washed twice with sterile phosphate-buffered saline (PBS; Gibco, Waltham, MA, USA) before treatment with 5 μL of calcein-AM and 20 μL of ethidium homodimer-1 in 10 mL PBS. Thereafter, the cells were incubated for 30 min at room temperature, and fluorescence images were immediately obtained using a confocal laser scanning microscope (LSM 510 META, Carl Zeiss Microscopy GmbH, Jena, Germany) in a dark room. The rate of cell viability was calculated in three random fields using ImageJ (ver. 1.53f accessed on 25 October 2020, National Institutes of Health, MD, USA, https://imagej.nih.gov/ij).

To investigate the differences in the cytoskeletal structures of keratinocytes based on the function of the five membranes, the cells attached to the five different surfaces were fixed with 2.5% glutaraldehyde for 1 h, permeabilized with 0.1% Triton X-100 (Sigma-Aldrich, St. Louis, MO, USA) in PBS for 15 min, then washed with 1X PBS and incubated with Alexafluor 488 Phalloidin (1:100, Invitrogen, St. Louis, MO, USA) at room temperature for 40 min in the darkroom. The samples were then treated with mounting media and DAPI (S36964, Invitrogen, Carlsbad, CA, USA). The stained cells were visualized using a confocal laser scanning microscope (LSM 510 META, Carl Zeiss Microscopy GmbH, Jena, Germany).

### 2.7. Statistics

Quantitative data are presented as mean ± standard deviation (SD). The significance of the differences between the two independent experiments was assessed using a Student’s *t*-test. *p* values below 0.05 were considered significant.

## 3. Results and Discussion

Figure 1c,d show the difference in bubble generation between the mixing of the PDMS solution using a Rushton impeller and hand-mixing (the existing method), and by the fabricated Rushton impeller in a coaxial mixer. With 3 min of impeller mixing, bubbles with a diameter smaller than 50 μm could be observed, which was 2.6 times more than those by hand-mixing of the PDMS solution. As the mixing time using the impeller increased from 1 to 5 min, the number of bubbles increased, and we confirmed that the bubbles smaller than 50 μm in diameter exponentially formed. Overall, there were no bubbles larger than 300 μm in diameter in the PDMS solution.

**Figure 2:**
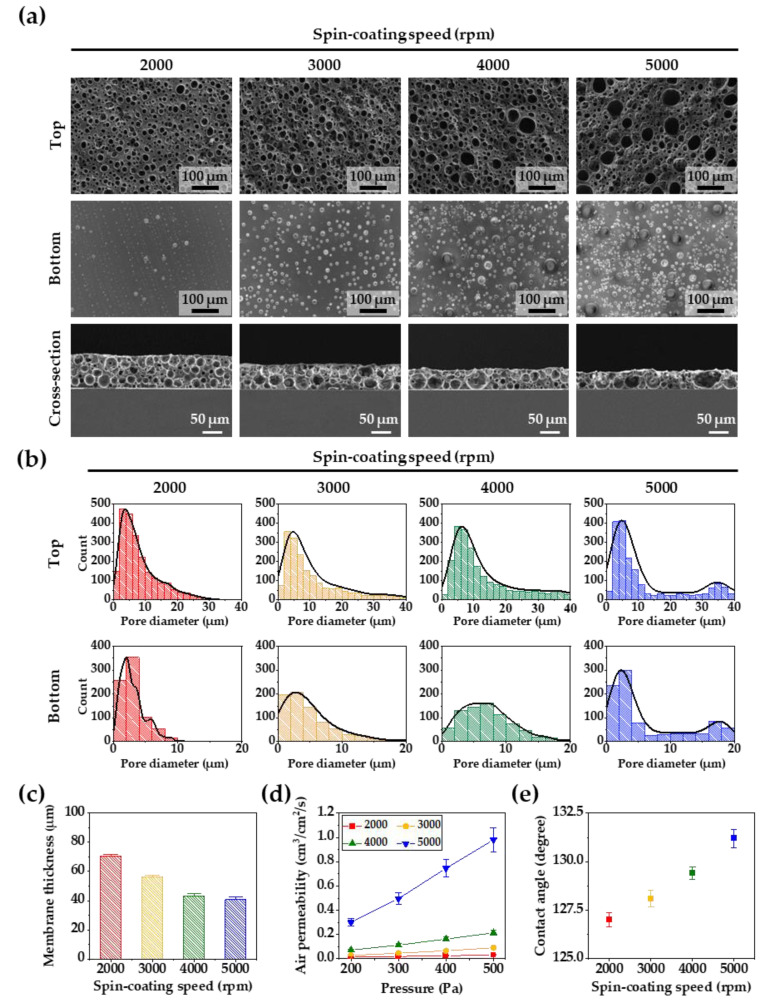
(**a**) SEM images of the top, bottom, and cross-section views of the porous PDMS membranes according to different spin-coating speeds. (**b**) The pore size distributions of the top and bottom sides of the porous PDMS membranes. (**c**) The membrane thickness at spin-coating speeds of 2000, 3000, 4000, and 5000 rpm. (**d**) Air permeability of the porous PDMS membranes as a function of pressures. (**e**) Water contact angles of the porous PDMS membranes surfaces according to different spin-coating speeds. For all the samples, the PDMS solution was mixed for 5 min using a modified Rushton impeller.

The effect of the Rushton impeller on micropore generation was investigated, as shown in Figure 2a,b, which show the top, bottom, and cross-section SEM images with a mixing time of 5 min of the porous membranes generated at spin-coating speeds of 2000, 3000, 4000, and 5000 rpm. In general, as the thickness of the PDMS membrane decreases, more micropores with diameters of 20 μm or less are created at the top and bottom of the membranes. However, in this experiment, small and large micropores at the top of the PDMS membranes increased as the thickness of the PDMS membranes decreased. At a spin-coating speed of 2000 rpm, small bubbles of 5 μm or less were dominant at the bottom of the PDMS membranes. As the thickness of the membranes increased, the micropores increased in size up to 20 μm, and small and large micropores were simultaneously formed at a spin-coating speed of 5000 rpm. In the cross-section image, more networking between small and large micropores was confirmed in the internal structure of the thinner PDMS membranes.

As shown in Figure 2c, the thickness of the PDMS membranes was controlled by the spin-coating speed. The thickness of the PDMS membrane decreased from 65 to 40 μm with an increase in the spin-coating speed from 2000 to 5000 rpm. In Figure 2d, the air permeability of the porous PDMS membranes is shown according to the membrane thickness. As the thickness of the membrane decreased, the air permeability increased. The PDMS membranes showed the best air permeability performance at the spin-coating speed of 5000 rpm. The air-permeable networking structure of the PDMS membranes play an important role in cell growth and tissue formation, resulting in enhanced formation of cell sheets. Figure 2e shows that the contact angle on the top surface of the porous PDMS membranes increased with a decrease in the membrane’s thickness and an increase in the membrane’s micropores. In the case of porosity-free PDMS, the contact angle was reported to be about 120 degrees [32], but in our experiment, the number of pores increased as the spin-coating speed increased, and many surface microstructures with hydrophobic properties were created. These results caused an increase in the hydrophobic specific surface area of the porous PDMS membrane, which resulted in an increase in the contact angle [33,34].

**Figure 3:**
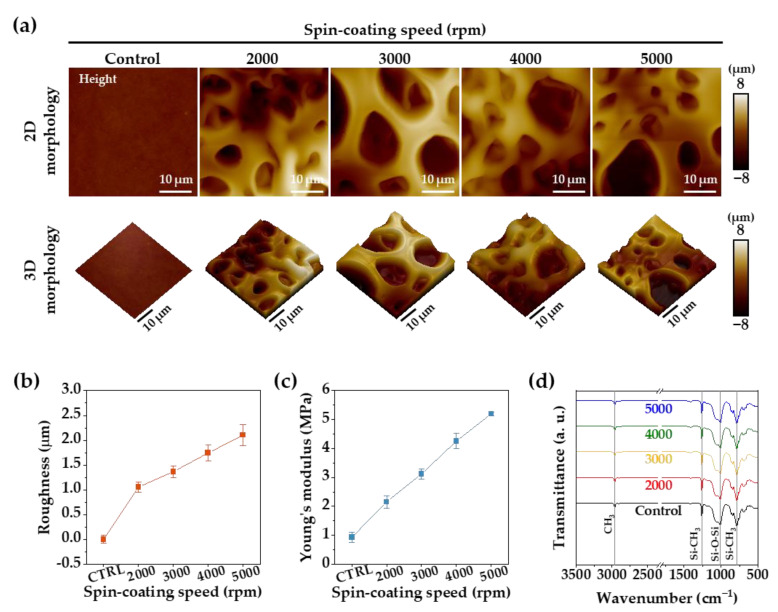
(**a**) The 2D and reconstructed 3D AFM images of the PDMS membranes. (**b**) Surface roughness of PDMS membranes obtained from the AFM analyses. (**c**) Young’s modulus of the PDMS membranes obtained from the AFM analyses. (**d**) ATR-FTIR spectra of the PDMS membrane surface.

Figure 3a shows the surface morphologies of the PDMS membranes (control, 2000, 3000, 4000, and 5000 rpm) visualized by AFM analysis. No roughness change was measured on the control membrane, but with a decrease in the porous membrane thickness, the roughness increased proportionally up to 2.1 ± 0.21 μm, as shown in Figure 3b. Additionally, in Figure 3c, the measured value of Young’s modulus linearly increased proportional to the spin-coating speed, up to a maximum of 5.2 ± 0.06 MPa. Small and large micropores of the porous PDMS membrane that were created at a spin-coating speed of 5000 rpm caused relatively larger increases in surface roughness and Young’s modulus than expected. Figure 3d indicates that no change in the chemical composition of the porous PDMS membrane was caused by the high-temperature fabrication process.

**Figure 4:**
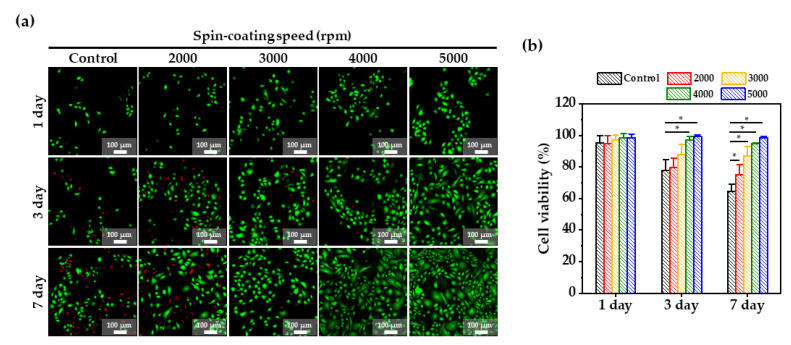
(**a**) Fluorescence images of a live and dead assay of keratinocyte cells cultured for 7 days on the PDMS membranes according to spin-coating speed; green—live cells; red—dead, and (**b**) Cell viability obtained from LIVE and DEAD assay at days 1, 3, and 7. *: *p*-value < 0.05.

Cell growth on the PDMS membranes was evaluated through the cell viability using a live/dead stain, as shown in Figure 4a,b. A high cell viability of over 90% was observed on all PDMS membranes on day 1. From day 3, the number of the live cells on the control, and 2000 and 3000 rpm membranes started to decrease, and on day 7, the number of live cells further decreased. However, the viability of cells on 4000 and 5000 membranes remained above 90%. It was shown that the biocompatibility and bioactivity of porous PDMS membrane enhanced the growth and proliferation of keratinocyte cells. As the PDMS membrane becomes thinner, more pores are created, which leads to an increase in roughness and Young’s modulus. The improvement in these mechanical properties creates a favorable environment for cell growth. In addition, the more air-permeable networking structure of the PDMS membrane helps cell growth by ensuring the smoother circulation of cell media. Therefore, the highest cell viability and proliferation were observed when the thickness of the PDMS membrane was 40 μm (spin-coating speed = 5000 rpm).

**Figure 5:**
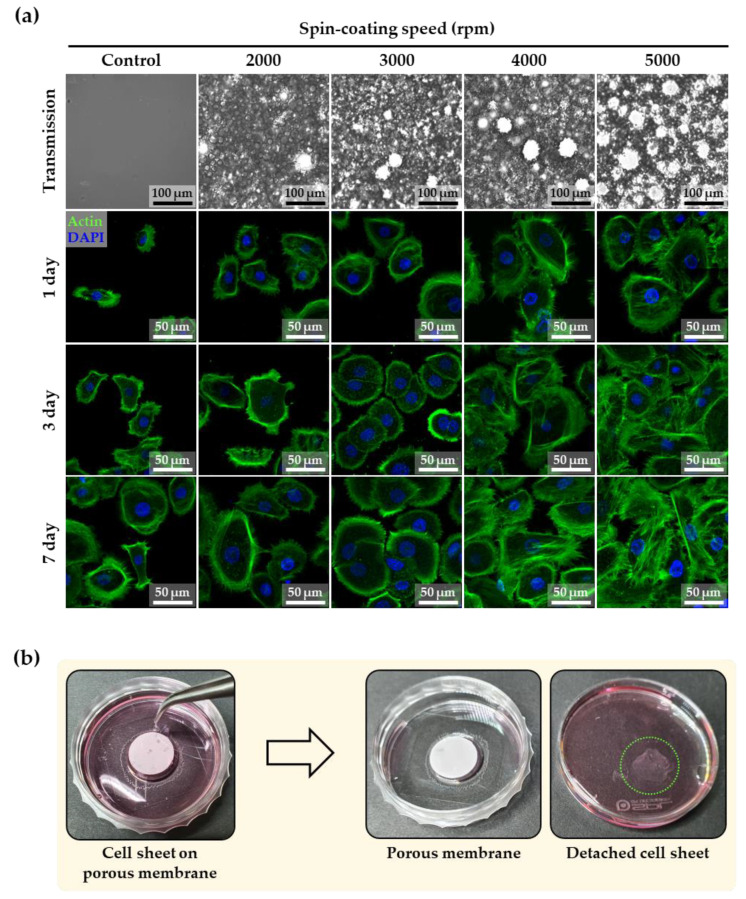
(**a**) Immunofluorescent images of actin and DAPI staining of keratinocyte cells on the PDMS membrane at days 1, 3, and 7 according to spin-coating speed. (**b**) Images before and after the detachment of cell sheet from the porous PDMS membrane (spin-coating speed of 5000 rpm) at day 7.

Figure 5a shows the day 1, 3, and 7 results of actin and DAPI staining immunofluorescence, which were used to evaluate the cytoskeleton and nuclei arrangement of normal human keratinocytes on PDMS membranes (control, and 2000, 3000, 4000, and 5000 rpm) for the formation of cell sheets. The fluorescent images showed that the cell staining was well-organized and -spread. As shown by the previous results, a large number of small and large micropores were generated from the PDMS membrane fabricated at the spin-coating speed of 5000 rpm, which had the highest values of roughness, Young’s modulus, contact angle, and air permeability. As shown by the day 7 actin staining results, the surface characteristics of the 5000 rpm membrane closely correlated with the most increased formation of actin stress fibers and migration of keratinocyte cells, resulting in the enhanced physical connection of actin stress fibers of the neighboring cells throughout the discontinuous adherent junctions. This correlation was proved by the enhanced cell sheet formation, as shown in Figure 5b. This figure confirms the intact detachment of the cell sheet from the porous PDMS membranes created at a spin-coating speed of 5000 rpm. This complete separation takes advantage of the hydrophobicity of PDMS. Plasma-treated PDMS initially showed a hydrophilic state, but returned to a hydrophobic state after seven days due to the transport of low molecular weight species from the bulk to the thermodynamically unstable hydrophilic surface [35,36,37]. This advantageous recovery gradually decreased the adhesion of the cell sheet on the porous PDMS membranes after seven days, allowing it to be easily separated from the porous membrane. This study demonstrates that a well-defined porous PDMS membrane can enhance the formation of a cell sheet to be used for tissue regeneration.

## 4. Conclusions

In this study, mixing a PDMS solution with a fabricated Rushton impeller in a co-axial mixer generated more bubbles than by hand-mixing. These bubbles affected the generation of a larger number of small and large micropores in the PDMS membranes with decreased membrane thickness. This porous membrane morphology had in higher values of roughness, Young’s modulus, contact angle, and air permeability. At day 7, the viability of cells on the porous PDMS membranes fabricated at a spin-coating speed of 5000 rpm was the highest (more than 98%) due to the internal networking structure and surface properties of that membrane, resulting in the enhanced physical connection of actin stress fibers from the neighboring cells throughout the discontinuous adherent junctions for cell sheet formation. The hydrophobicity recovery characteristic of PDMS facilitated the intact detachment of the cell sheet from the porous PDMS membrane. Based on our results, well-defined porous PDMS membranes have considerable potential for enhancing the formation of cell sheets for use in regenerative medicine.

## Data Availability

Not applicable.
